# Cimetidine modulates the antigen presenting capacity of dendritic cells from colorectal cancer patients

**DOI:** 10.1038/sj.bjc.6600233

**Published:** 2002-04-22

**Authors:** T Kubota, H Fujiwara, Y Ueda, T Itoh, T Yamashita, T Yoshimura, K Okugawa, Y Yamamoto, Y Yano, H Yamagishi

**Affiliations:** Department of Digestive Surgery, Kyoto Prefectural University of Medicine, 465 Kajii-cho, Kawaramachi-hirokoji, Kamigyo-ku, Kyoto, 602-0841, Japan

**Keywords:** dendritic cell, H_2_ receptor antagonist, cimetidine, colorectal cancer

## Abstract

Cimetidine, a H_2_ receptor antagonist, has been reported to improve survival in gastrointestinal cancer patients. These effects have largely been attributed to the enhancing effects of cimetidine on the host's antitumour cell-mediated immune response, such as inhibition of suppressor T lymphocyte activity, stimulation of natural killer cell activity and increase of interleukin-2 production from helper T lymphocytes. We conducted an *in vitro* study on the effects of cimetidine on differentiation and antigen presenting capacity of monocyte-derived dendritic cells from advanced colorectal cancer patients and normal controls. As a result, an investigation of expression of surface molecules associated with dendritic cells by flow cytometric analyses showed that cimetidine had no enhancing effect on differentiation of dendritic cells from cancer patients and normal controls. An investigation of [^3^H]thymidine incorporation by allogeneic mixed lymphocyte reactions revealed that cimetidine increased the antigen presenting capacity of dendritic cells from both materials. Moreover, a higher antigen presenting capacity was observed in advanced cancer patients compared to normal controls. These effects might be mediated via specific action of cimetidine and not via H_2_ receptors because famotidine did not show similar effects. Our results suggest that cimetidine may enhance the host's antitumour cell-mediated immunity by improving the suppressed dendritic cells function of advanced cancer patients.

*British Journal of Cancer* (2002) **86**, 1257–1261. DOI: 10.1038/sj/bjc/6600233 www.bjcancer.com

© 2002 Cancer Research UK

## 

Cimetidine, a histamine type 2 (H_2_) receptor antagonist, widely used to treat peptic ulcers, has also been shown to have clinical benefits in cancer patients. It was first reported in 1988 that a postoperative course of cimetidine improved survival in gastric cancer patients (Tonnesen *et al*, 1988). Since then, several studies have been published showing major survival advantages in gastrointestinal cancer patients treated with cimetidine (Adams and Morris, 1994; Matsumoto, 1995; Kelly *et al*, 1999). Many studies on the mechanisms of this action have indicated that the antitumour effects of cimetidine might be due to a direct inhibitory effect on tumour growth (Adams and Morris, 1994; Adams *et al*, 1994; Reynolds *et al*, 1996), cell-mediated immunomodulation (Osband *et al*, 1981; Hellstrand and Hermodsson, 1986; Gifford and Tirberg, 1987), or inhibition of cancer cell metastases (Kobayashi *et al*, 2000). The mechanisms proposed for cell-mediated immunomodulation of cimetidine include inhibition of suppressor T lymphocyte activity (Osband *et al*, 1981), stimulation of natural killer (NK) cell activity (Hellstrand and Hermodsson, 1986), and increase of interleukin-2 (IL-2) production in helper T lymphocytes (Gifford and Tirberg, 1987).

Dendritic cells (DC), which are potent antigen presenting cells capable of priming naive T lymphocytes and subsequently inducing cytotoxic T lymphocytes (CTL) by stimulation of Th1 type immune response, play a central role in cell-mediated immunity (Janeway *et al*, 1997; Banchereau and Steinman, 1998). With the recent development of culture methods for propagating DC on a large scale from human peripheral blood mononuclear cells (PBMC) (Caux *et al*, 1992; Sallusto and Lanzavecchia, 1994), vaccination aimed at efficient production of CTL with tumour-antigen-loaded DC represents a potentially powerful strategy to induce tumour rejection (Young and Inaba, 1996; Nestle *et al*, 1998). Moreover, since it has also been reported that DC stimulate NK cell activity (Fernandez *et al*, 1999; Yu *et al*, 2001), DC should be considered to be associated intimately with not only the production of CTL but also with the whole process of antitumour cell-mediated immunity. However, there is little published information regarding the influence of cimetidine on DC function.

Based on the above-mentioned findings, it was reported recently that histamine inhibits the secretion of human interleukin-12 (IL-12) via H_2_ receptors expressed on monocytes (precursors of DC), and these effects of histamine can be reversed by H_2_ receptor antagonists such as cimetidine (Elenkov *et al*, 1998; Tineke *et al*, 1998).

In the present study, we assumed that cimetidine might have some influence on monocyte-derived DC functions via H_2_ receptors and investigated the effects of cimetidine on *in vitro* (1) differentiation, (2) antigen presenting capacity, and (3) IL-12 production of monocyte-derived DC from colorectal cancer patients and normal controls.

## MATERIALS AND METHODS

### Patients and controls

The study has been carried out with the ethical committee approval. Ten patients (four men and six women) with advanced colorectal cancer, aged 28–65 years (means±s.d.; 50.6±11.5 years) were studied (Table 1

**Table 1:**
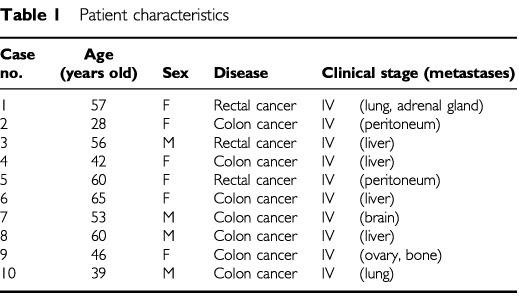
Patient characteristics

). All tumours were classified as stage IV according to tumour-node-metastasis (TNM). All patients had received chemotherapy and/or operation and had the interval of more than 4 weeks prior to the present study. Their leukocyte numbers were within normal limits. The control subjects consisted of 10 age-matched healthy volunteers (eight men and two women). All patients and all healthy volunteers were free from infection and other complications at the time of study.

### Media and reagents

RPMI 1640 supplemented with 4 mM L-glutamine and NaHCO_3_ (Nikken, Kyoto, Japan), 100 IU ml^−1^ penicillin and 100 μg ml^−1^ streptomycin (Sigma, UK), 50 μM 2-mercaptoethanol, and 10% heat-inactivated foetal calf serum (FCS) was used as culture medium throughout the experiments. Human recombinant granulocyte macrophage-colony stimulating factor (GM-CSF) and interleukin-4 (IL-4) were kindly provided by Kirin Brewery (Gunma, Japan) and Genzyme (Minneapolis, MN, USA), respectively. Cimetidine and famotidine were kindly provided by Smith Kline Beecham, Japan (Tokyo, Japan) and Yamanouchi (Tokyo, Japan), respectively.

### Generation of DC

PBMC were obtained from 10 patients with advanced colorectal cancer by leukapheresis using Blood Cell Separator CS-3000™ (Baxter, Deerfield, IL, USA) after informed consent was obtained. As control subjects, PBMC from healthy volunteers were prepared by density gradient centrifugation on Ficoll-Hypaque Plus (Pharmacia Biotech, Sweden). Interphases were harvested and washed twice with RPMI 1640 at low speed to remove platelets. Monocytes were separated from these PBMC by plastic dish adhesion for 2 h at 37°C in a 5% CO_2_ atmosphere and were further incubated for 7 days at 37°C in culture medium supplemented with 500 U ml^−1^ of GM-CSF and 500 U ml^−1^ of IL-4. These monocyte-derived DC were used for surface analysis and mixed lymphocyte reaction.

### Flow cytometric analyses

At day 0 of PBMC incubation, 1.0 or 10.0 μg ml^−1^ of cimetidine or 0.1 or 1.0 μg ml^−1^ of famotidine was added to the culture medium, and at day 7, expression of cell surface molecules associated with DC differentiation was analysed using FACScan (Becton Dickinson, Mountain View, CA, USA) and Cell Quest software. The dose of each H_2_ receptor antagonist (1.0 μg ml^−1^ of cimetidine, 0.1 μg ml^−1^ of famotidine) was based on the EC_50_, which denotes the serum concentrations of the drug necessary to inhibit the pentagastrin-stimulated secretion of acid by 50% (Feldman and Burton, 1990). Direct immunofluorescence cell staining was performed using PE-conjugated anti-CD80 monoclonal antibodies (mAb) (Phar Mingen, San Diego, CA, USA) and PE-conjugated isotype control antibodies (Becton Dickinson, San Jose, CA, USA). Indirect immunofluorescence was performed by staining with unconjugated anti-CD86 mAb (Ancell, Bayport, MN, USA), CR3/43 mAb for HLA-DP/DQ/DR (DAKO A/S, Denmark) and isotype-matched control mAb followed by PE-conjugated F (ab′) two fragments of rabbit anti-mouse IgG/FITC (DAKO A/S, Denmark) as secondary antibody. Ten thousand cells were analysed by flow cytometry and the results were presented as the ratio of the mean channel with cimetidine or famotidine divided by the mean channel without them.

### Allogeneic mixed lymphocyte reactions (allo MLR)

DC of colorectal cancer patients and healthy volunteers were generated from PBMC as described above. Allogeneic T lymphocytes were isolated from PBMC of a single healthy volunteer by nylon fibre non-adherence using T lymphocyte isolation columns (Nylon Fiber Column T, Wako, Japan). The stimulator cell fractions (DC) were irradiated with 30 Gy. After extensive washing different numbers of stimulators were added to the culture wells containing a fixed amount of T lymphocytes (10^5^ well^−1^) so that the final stimulator to responder ratio (R/S ratio) ranged from 20/1 to 80/1. At day 0 of coculture, 1.0 μg ml^−1^ of cimetidine or 0.1 μg ml^−1^ of famotidine was added to the culture medium. During the last 8 h of 5 days of culture, 1 μCi well^−1^ [^3^H]thymidine (Amersham Pharmacia Biotech, UK) was added. Cells were then harvested and radioactivity of [^3^H]thymidine was measured with a scintilation counter (Packard, Meriden, CT, USA). The responses of allogeneic T lymphocytes were expressed as mean radioactivity (c.p.m.) of [^3^H]thymidine incorporated per well. The stimulation index (S.I) was used to quantify the frequency of allogeneic T lymphocyte proliferation. The S.I was expressed as the ratio of c.p.m. with cimetidine or famotidine to c.p.m. without them.

### IL-12 assay

DC from colorectal cancer patients and healthy volunteers, and allogeneic T lymphocytes from a single healthy volunteer were cocultured at R/S ratio of 10/1 in the presence of 1.0 μg ml^−1^ of cimetidine. After 5 days of culture, supernatants were centrifuged to remove residual cells and stored in −20°C until use. IL-12 p70 heterodimer levels in the supernatants were measured by sandwich type enzyme-linked immunosorbent assay (ELISA) (Immunotech, France) according to the manufacture's instructions. All tests were performed in duplicate. The sensitivity levels of the ELISA assays were 5 pg ml^−1^.

### Statistical analysis

Results were presented as means±standard deviation (s.d.). Student's *t-*test was applied to test significant differences and a *P* value of <0.05 was considered to indicate statistical significance. All tests were two-tailed.

## RESULTS

### Effect of cimetidine and famotidine on DC differentiation

Flow cytometry was used to investigate the effect of cimetidine and famotidine on the differentiation of DC. Expression of MHC–class II, CD80 and CD86 was analysed and viability of differentiated cells was measured.

As a result, no enhancing effect of cimetidine on DC differentiation was found. As shown in Table 2

**Table 2:**
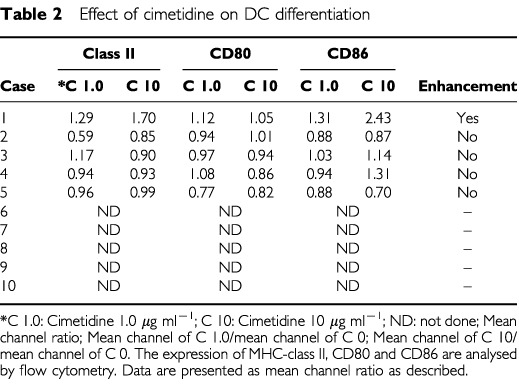
Effect of cimetidine on DC differentiation

, cimetidine slightly increased the expression of surface molecules only in Case 1, but not in the other cases tested. The analysis was stopped in Case 6 because positive data were not found after Case 2. Famotidine showed no effects in any cases tested (data not shown). Similar results were obtained in healthy volunteers (data not shown) or at increasing concentrations (5-, 10-, 50-fold) of each H_2_ receptor antagonist (data not shown). These results were substantiated by the fact that both cimetidine and famotidine did not enhance the viability of differentiated cells (data not shown).

### Effect of cimetidine and famotidine on antigen presenting capacity of DC

Allo MLR was carried out to investigate the effect of cimetidine and famotidine on the antigen presenting capacity of DC, and [^3^H]thymidine incorporation of allogeneic T lymphocytes was measured. Monocyte-derived DC generated as described in Materials and Methods were cocultured with allogeneic T lymphocytes from a single healthy volunteer in the presence of 1.0 μg ml^−1^ cimetidine or 0.1 μg ml^−1^ famotidine.

**Figure 1:**
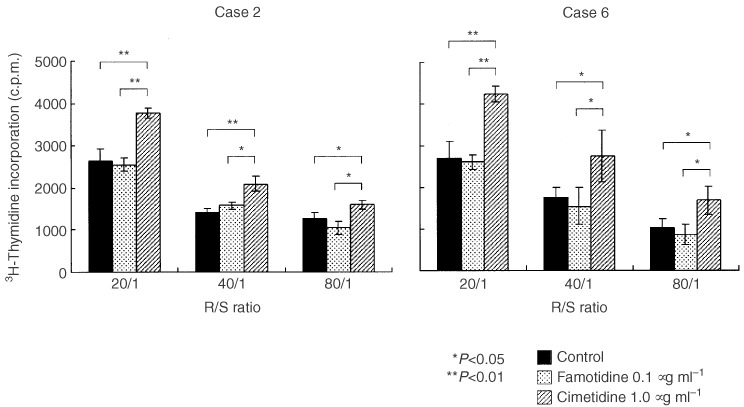
Antigen presenting capacity of two typical cases (Case 2 and Case 6). Data are presented as amounts (c.p.m.) of [^3^H]thymidine incorporation.

**Figure 2:**
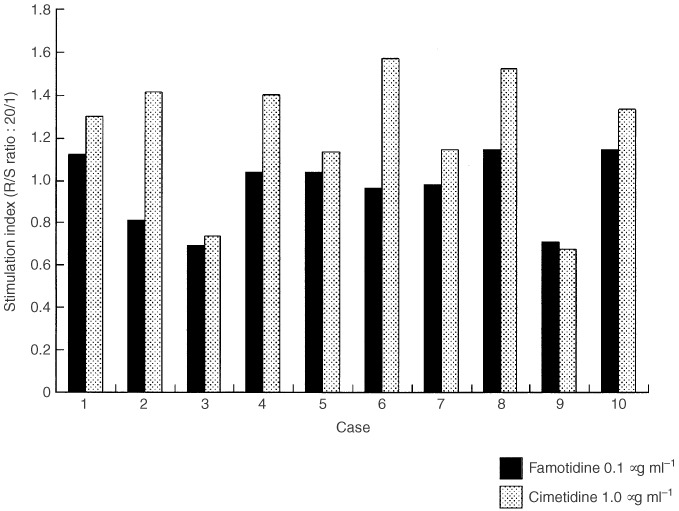
Comparison of S.I of each case with cimetidine and famotidine at R/S ratio of 20/1.

**Figure 3:**
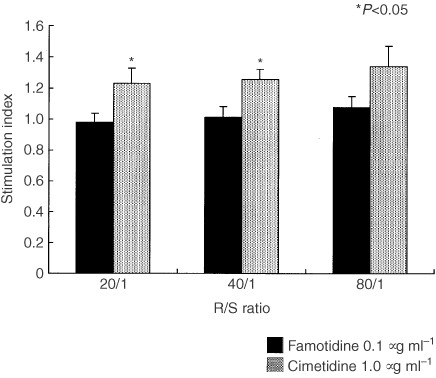
Comparison of mean S.I with cimetidine and famotidine at R/S ratio of 20/1 ∼ 80/1.

**Figure 4:**
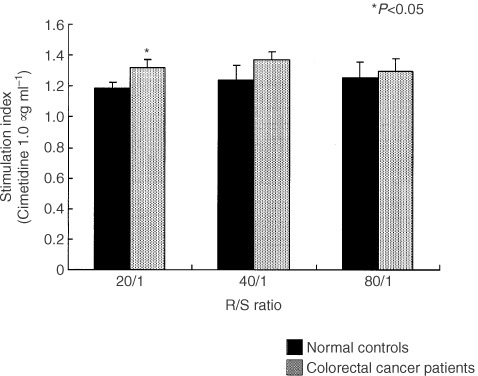
Comparison of mean S.I with colorectal cancer patients and normal controls at R/S ratio of 20/1 ∼ 80/1.

As a result, in eight out of 10 colorectal cancer patients, cimetidine obviously increased [^3^H]thymidine incorporation of allogeneic T lymphocytes compared to famotidine (Figure 1

). In two typical cases (Case 2 and Case 6), cimetidine increased significantly and constantly [^3^H]thymidine incorporation at each R/S ratio of 20/1 to 80/1 (Figure 2

). Moreover, mean S.I of cimetidine at each R/S ratio in all cases was significantly higher than that of famotidine (Figure 3

). In a comparison between colorectal cancer patients and normal controls, cimetidine showed higher increases in the former than in the latter (*P*=0.048 at 20/1) (Figure 4

). On the other hand, famotidine did not show any increase both in cancer patients and normal controls (data not shown).

### Effect of cimetidine on IL-12 production of DC

IL-12 concentrations of the supernatants obtained by coculture of DC with allogeneic T lymphocytes were measured to evaluate the effect of cimetidine on DC function.

IL-12 production of DC in colorectal cancer patients (*n*=7) was slightly lower than in normal controls (*n*=4). However, although cimetidine did not affect IL-12 production of DC in normal controls, it tended to increase IL-12 production in colorectal cancer patients up to the level of normal controls (*P*=0.383) (Table 3

**Table 3:**
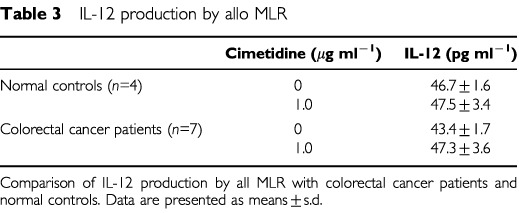
IL-12 production by allo MLR

).

## DISCUSSION

The clinical effectiveness of cimetidine against gastrointestinal malignancies has been reported and various mechanisms of action have been proposed. In this study, we discovered for the first time the possibility that cimetidine may increase the antigen presenting capacity of monocyte-derived DC from advanced colorectal cancer patients although it does not enhance their differentiation. These results suggest that cimetidine enhances antitumour cell-mediated immune response by stimulating DC to activate Th1 type immune response and subsequent CTL induction. Gifford and Tirberg (1987) demonstrated that cimetidine increased IL-2 production from mitogen-activated murine spleen cells and this effect might be due to stimulation of helper T lymphocytes by antigen presenting cells. The present results support their findings.

Our observation that cimetidine increased the antigen presenting capacity of DC from colorectal cancer patients compared to DC from normal controls implies improvement of suppressed DC function in immunosuppressed cancer patients by cimetidine. Dysfunction of DC in advanced cancer patients is predictable and Ninomiya *et al* (1999) have demonstrated that DC from hepatocellular carcinoma had significantly lower capacity to stimulate allogeneic T lymphocytes in allo MLR compared to DC from normal controls. The stimulatory effect of cimetidine on T lymphocytes is well-known (Rocklin, 1976; Gifford *et al*, 1980), however, it is unlikely that the difference of [^3^H]thymidine incorporation between cancer patients and normal controls is caused only by the effect of cimetidine on T lymphocytes because T lymphocytes from a single healthy volunteer were used as responders in allo MLR.

To confirm the hypothesis that cimetidine gives a direct action to DC themselves and improve the antigen presenting capacity of DC from colorectal cancer patients, we measured IL-12 in the supernatants of allo MLR. IL-12 is well known as a cytokine that is produced by DC responding to antigen stimulation and acts CD4+ helper T lymphocytes to induce Th1-type immune responses (Banchereau and Steinman, 1998). The present results indicate that IL-12 was produced from DC stimulated by allogeneic T lymphocytes and cimetidine might improve the suppressed DC function of colorectal cancer patients. Therefore, we conclude that the increase of [^3^H]thymidine incorporation in allo MLR may be due to some effects of cimetidine on not only T lymphocytes but also DC themselves or the interaction between DC and T lymphocytes.

On the other hand, famotidine, another H_2_ receptor antagonist, did not show the same effects as cimetidine. Because famotidine behaves as a specific H_2_ receptor antagonist with a molar potency four to eight times greater than that of cimetidine (Peden *et al*, 1982; Feldman and Burton, 1990), it is natural that famotidine should show equal or greater effects if the effect of cimetidine is mediated via H_2_ receptors. In this regard, cimetidine has been reported to have better cell-mediated immunomodulation (e.g. proliferation and cytotoxicity of lymphocytes) or histamine (or H_2_ receptor)-dependent inhibitory effects on tumour growth than other H_2_ receptor antagonists such as famotidine and ranitidine, and the differences between cimetidine and other H_2_ receptor antagonists might be due to their structures and/or affinities to H_2_ receptors (Morris and Adams, 1995; Lawson *et al*, 1996). Kobayashi *et al* (2000) showed that cimetidine can block the adhesion of colorectal cancer cells to the endothelial cells, suppressing the metastases of cancer cells. They also considered that these actions of cimetidine are not mediated via H_2_ receptors, because other H_2_ receptor antagonists, famotidine and ranitidine, did not show a similar effect. While it remains unclear whether H_2_ receptors are expressed on DC or not, the effect of cimetidine on the antigen presenting capacity of DC appears to arise because of cimetidine-specific actions.

Although it remains unclear whether or not the modulating effects of cimetidine on DC function observed in our investigation *in vitro* have clinically substantial meanings, clinical effectiveness of cimetidine against gastrointestinal malignancies are considered to be due to the total of immunological and non-immunological actions of cimetidine.

Finally, both tumour-antigen-specific and non-specific immunosuppression have been observed in the tumour-bearing host (Roth, 1983; Ninomiya *et al*, 1999). Therefore, immunostimulation offers theoretical benefits for immunotherapy. Further investigation into DC functions is promising in the search for more clinically effective tumour-antigen-specific immunotherapy and also for the elucidation, of immunosuppressive mechanisms in tumour-bearing hosts.
